# Supplementary material for: A high-resolution mRNA expression time course of embryonic development in zebrafish
Source: eLife. 2017 Nov 16;6:e30860. doi: 10.7554/eLife.30860 (PMC5690287; doi:10.7554/eLife.30860)
Supplement: Supplementary file 6. [file elife-30860-supp6.zip › biolayout-clusters-files/Cluster089-genes.html]

Cluster089


# Cluster089: Genes

| Ensembl ID | Gene Name | Chr | Start | End | Biotype |
| --- | --- | --- | --- | --- | --- |
| ENSDARG00000014465 | arhgef25b | 6 | 39258634 | 39278385 | protein\_coding |
| ENSDARG00000061140 | colec12 | 2 | 31399372 | 31451940 | protein\_coding |
| ENSDARG00000026369 | dbi | 9 | 907950 | 915377 | protein\_coding |
| ENSDARG00000052178 | eif2s1b | 20 | 51291147 | 51310045 | protein\_coding |
| ENSDARG00000054619 | fras1 | 5 | 38237018 | 38486615 | protein\_coding |
| ENSDARG00000101892 | ino80e.1 | 16 | 10531934 | 10537997 | protein\_coding |
| ENSDARG00000012824 | itga3b | 12 | 5552390 | 5628232 | protein\_coding |
| ENSDARG00000011724 | marveld1 | 13 | 39251340 | 39365301 | protein\_coding |
| ENSDARG00000071215 | palm3 | 6 | 7742072 | 7963434 | protein\_coding |
| ENSDARG00000068973 | pdlim4 | 21 | 43743222 | 43806335 | protein\_coding |
| ENSDARG00000055064 | prdx5 | 21 | 26620617 | 26628034 | protein\_coding |
| ENSDARG00000014101 | pyroxd2 | 13 | 40158833 | 40190475 | protein\_coding |
| ENSDARG00000039652 | si:dkey-235d18.5 | 20 | 52778916 | 52920080 | protein\_coding |
| ENSDARG00000058381 | zgc:171775 | 23 | 10211572 | 10230063 | protein\_coding |
